# In Vitro Pharmacokinetic Behavior of Antiviral 3-Amidinophenylalanine Derivatives in Rat, Dog and Monkey Hepatocytes

**DOI:** 10.3390/biomedicines11030682

**Published:** 2023-02-23

**Authors:** Katalin Lányi, Katalin Monostory, Torsten Steinmetzer, Ákos Jerzsele, Erzsébet Pászti-Gere

**Affiliations:** 1 Department of Food Hygiene, University of Veterinary Medicine, István utca 2, H-1078 Budapest, Hungary; 2 Research Centre for Natural Sciences, Institute of Enzymology, Magyar Tudósok 2, H-1117 Budapest, Hungary; 3 Department of Pharmacy, Institute of Pharmaceutical Chemistry, Philipps University Marburg, Marbacher Weg 6, G-35032 Marburg, Germany; 4 Department of Pharmacology and Toxicology, University of Veterinary Medicine, István utca 2, H-1078 Budapest, Hungary; 5 National Laboratory of Infectious Animal Diseases, Antimicrobial Resistance, Veterinary Public Health and Food Chain Safety, University of Veterinary Medicine, István utca 2, H-1078 Budapest, Hungary

**Keywords:** antiviral, cynomolgus monkey, 3-amidinophenylalanine, depletion rate, matriptase, TMPRSS2, coronavirus, rat, dog, intrinsic clearance

## Abstract

Type II transmembrane serine proteases represent pharmacological targets for blocking entry and spread of influenza or coronaviruses. In this study, the depletion rates of the 3-amidinophenylalanine (3-APhA)-derived matriptase/TMPRSS2 inhibitors MI-463, MI-472, MI-485 or MI-1900 were determined by LC-MS/MS measurements over a period of 300 min using suspensions of rat, dog and cynomolgus monkey primary hepatocytes. From these in vitro pharmacokinetic (PK) experiments, intrinsic clearance values (Cl_int_) were evaluated, and in vivo pharmacokinetic parameters (hepatic clearance, hepatic extraction ratio and bioavailability) were predicted. It was found that rat hepatocytes were the most active in the metabolism of 3-APhA derivatives (Cl_int_ 31.9–37.8 mL/min/kg), whereas dog and monkey cells displayed somewhat lower clearance of these compounds (Cl_int_ 6.6–26.7 mL/min/kg). These data support elucidation of important PK properties of anti-TMPRSS2/anti-matriptase 3-APhAs using mammalian hepatocyte models and thus contribute to the optimization of lead compounds.

## 1. Introduction

Both matriptase and transmembrane serine protease 2 (TMPRSS2) belong to the type II transmembrane serine proteases (TTSPs), and these membrane-anchored enzymes are involved in several physiological and pathological processes [1]. Excessive TTSP activity can be correlated with the formation of several disorders, thus specific inhibition of matriptase- and TMPRSS2-caused proteolysis with 3-amidinophenylalanine (3-APhA) derivatives can be an effective pharmacological option to alleviate tumor formation and metastatic potential [2], to counteract cartilage damage [3] and to restore hepcidin-mediated tipped iron homeostasis [4].

Moreover, these enzymes appear to be responsible for proteolytic activation of viral glycoproteins, thus facilitating certain viruses’ replication and spread. TMPRSS2 was reported to cleave hemagglutinin (HA) of avian and mammalian influenza A viruses (IAV) with a monobasic cleavage site in mice [5]. Other viruses such as Middle East respiratory syndrome- (MERS-) and severe acute respiratory syndrome (SARS) coronaviruses (CoV) also require activation of spike (S) glycoprotein for early entry into the cells via host TMPRSS2-mediated proteolytic cleavage [6,7]. Matriptase, which is highly expressed in a membrane-bound form, can also cleave HA precursor [8]. 

A new type of coronavirus, SARS-CoV-2 emerged in 2019 in China and often caused serious pneumonia (COVID-19) in humans, becoming rapidly pandemic. Vaccination is one of the main therapeutic measures to limit the spread of viral infection; however, the appearance of mutated variants of concern (VOCs) necessitates the clinical application of antivirals such as the direct-acting nucleotide prodrug remdesivir [9,10] and the nucleoside molnupiravir as well as the orally available inhibitor of the viral M ^pro^ protease inhibitor Paxlovid (combination of nirmatrelvir and ritonavir) [11,12]. The non-selective serine protease inhibitors, camostate mesylate originally used in the therapy of postoperative reflux esophagitis and acute exacerbations of chronic pancreatitis and antifibrinolytic aprotinin, were repurposed as drug candidates for COVID-19; however, camostate failed during clinical testing [13]. So far, host-directed small molecule peptidomimetics have been developed, such as the sulfonylated 3-APhA derivatives [14,15] used in this study as well as several ketobenzothiazole (ktb)-based inhibitors [16,17] to impede the proteolytic activation of the SARS-CoV-2 S protein. For improving the stability and potency, the structure of the latter compound type was optimized. The most promising inhibitor, N-0385 (Ms-Gln-Phe-Arg-kbt), possesses an IC50 value of 1.9 ± 1.4 nM. It was also found that N-0385 was effective in inhibition of SARS-CoV-2 infection in human lung epithelium-derived human airway Calu-2 cells and in a K18-human ACE2 transgenic mouse model [18].

Several animal species have been identified to be susceptible to SARS-CoV-2 infections [19]. Although the disease seems to be of zoonotic origin, transmission of the pathogen from humans to animals was also reported. Dogs show some susceptibility to infection, possibly due to the presence of angiotensin-converting enzyme 2 (ACE2), as in humans with similar clinical signs of the disease, and it was reported that dogs could also produce positive virological tests and that virus transmission was possible from humans to dogs under natural conditions [20]. Non-human primates (NHP) have high susceptibility to SARS-CoV-2 infection, and their symptoms resemble human pathological conditions to a great extent. Type II pneumocyte hyperplasia and alveolar fibrosis were mainly found in lung tissue from cynomolgus macaques, proving that these animals can be successfully infected by airborne SARS-CoV-2 [21]. On the other hand, animal models enable in-depth characterization of SARS-CoV-2 pathophysiology and can contribute to the development and preclinical investigations of antiviral therapies. Severity of respiratory disorders in cynomolgus macaques was correlated well with the ages and underlying diseases (diabetes and hyperlipidemia) of the animals infected experimentally with coronavirus [22]. Other mammalian models including hamsters, ferrets, and mice with hACE2 Tg were found to be also susceptible to SARS-CoV-2, showing respiratory transmission; however, sex- and age-related severity differences can be mainly seen in cynomolgus monkey models [23]. 

The tested 3-APhA-derived inhibitors of trypsin-like serine proteases can serve as start structures for the development of potential antiviral drugs. The suppression of host cell proteases can be favorable versus classical antiviral therapies due to the low incidence of drug resistance [24,25]. The application of these drug candidates can hinder the entry of SARS-CoV-2 via preventing TMPRSS2-mediated activation of S protein necessary for the attachment of virus particles to the host cells through surface ACE2 receptors [26]. Combinative administration of furin and TMPRSS2 inhibitors due to their different cleavage sites of S (S1/S2 and S2′) was proven to have excellent antiviral properties in airway epithelial cells infected with SARS-CoV-2; therefore, their simultaneous application can lead to a more effective synergistic effect. This was the first study in which peptidomimetic sulfonylated 3-APhA-based MI compounds including MI-432 and MI-1900 were used to effectively decrease SARS-CoV-2 virus titer [27]. 

Until now, no predicted pharmacokinetic (PK) data have been available for 3-APhA-based inhibitors after their interaction with hepatocytes of different animal species. In this study, depletion % of the applied inhibitors (MI-463, MI-472, MI-485 and MI-1900) was determined in primary rat, dog and cynomolgus monkey hepatocytes using LC-MS/MS measurements. Interspecies comparisons of depletion of inhibitors over the chosen time window (0–300 min) were also performed to assess the impact of certain chemical structures and the effect of species on biotransformation rates of these 3-APhA derivatives. In addition, in vitro intrinsic clearance (Cl_int_) values were evaluated for each MI compound, and in vivo PK parameters, such as predicted Cl (Cl_H_), hepatic extraction ratio (E_H_) and bioavailability (F), were predicted.

## 2. Materials and Methods

### 2.1. Preparation of Primary Hepatocyte Suspensions

Cryopreserved rat, dog and cynomolgus monkey primary hepatocytes were obtained from Primacyt (Primacyt, Schwerin, Germany). Hepatocytes were thawed using cryopreserved hepatocyte thawing medium (Primacyt, Schwerin, Germany), and cells were centrifuged (10 min, 100× *g*, 20 °C) to remove supernatants. The viability of hepatocytes was examined by the Trypan blue exclusion method, and it was confirmed that more than 90% of the cells were alive. Cell suspensions were then diluted with serum-free maintenance medium (Primacyt, Schwerin, Germany) after cell counting in Bürker chamber to the appropriate concentration (original concentration of the viable hepatocytes was 10^7^ cells/mL). 

### 2.2. Incubation of Primary Hepatocytes with Inhibitors

**Figure 1:**
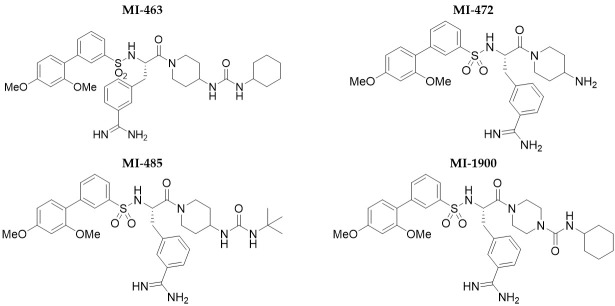
Structures of the matriptase/TMPRSS2 inhibitors applied in this study.

Stock solutions of MI-463, MI-472, MI-485 and MI-1900 (10 mM) in dimethyl-sulfoxide were kept at −20 °C. The structures of the inhibitors are shown in Figure 1. Time courses of the unchanged amount of the inhibitors (in %) after incubation with hepatocytes were measured. Each compound was incubated in cell suspension (at the concentration of 5 × 10^5^ cells/mL in 6-well plates) at constant and low-speed shaking at 37 °C in a humid atmosphere containing 5% CO_2_. The solutions of the inhibitors at 10 µM were prepared with maintenance medium freshly from the stock prior to each experiment (DMSO concentration did not exceed 0.1% in the final solutions). Cells were incubated with serum-free maintenance medium either without or with each selected MI for 5 h. At various time points (0, 30, 60, 120, 180, and 300 min), the incubation mixtures were sampled (aliquots: 0.15 mL) and terminated by the addition of 0.1 mL ice-cold acetonitrile. The cell debris was separated by centrifugation, and the supernatants were analyzed by LC-MS/MS for quantitation of the remaining amount of the inhibitors. 

### 2.3. Determination of Unchanged Parent Compound Concentrations

For the LC-MS/MS investigations, 120 μL from the incubation mixture samples obtained as described above was removed and mixed with 315 μL chilled acetonitrile and 15 μL triphenylphosphate solution (TPP; 12000 ng/mL in acetonitrile) used as internal standard for the quantitation. After vortex mixing, the samples were centrifuged at 4 °C and 13300 RPM for 10 min. The supernatant was filtered through a PVDF syringe filter of 0.22 μm pore diameter, then transferred to chromatographic vials.

The LC-MS/MS instrument used for analyzing the MI compounds was a Shimadzu LCMS 8030+ system operated with an electrospray ion source (ESI) in positive multiple reaction monitoring (MRM) mode. A Phenomenex Kinetex C18 EVO 100 × 4.6 mm ID (2.6 µm particle size) column equipped with a 40 × 2 mm C18 guard column was used for the chromatographic separation. Gradient elution was used with eluents “A”: 50 mM ammonium acetate in water (pH 5 set by acetic acid) and “B”: 0.1 *v/v%* formic acid in acetonitrile. The flow rate was 0.4 mL/min, a chromatographic run took 8 min. The column oven was set to 30 °C, and the autoinjector’s temperature was at 6 °C. The injected volume was 10 µL. Other MS parameters were as follows: interface 4.5 kV; interface temperature 350 °C; desolvation line 300 °C; heat block 400 °C; detector 1.78 kV, nebulizing gas (N_2_) 3 liters/min, drying gas (N_2_) 15 liters/min; collision gas (Ar) 230 kPa.

### 2.4. Assessment of Pharmacokinetics Parameters

The intrinsic clearance for hepatocytes (Cl_int_) [mL (min 5 × 10^5^ cells) ^−1^] was calculated from the decrease in the concentration of MI compounds as follows: *Cl*_int_ = $\beta =\frac{\ln2}{t_{1/2}}$ [28] For scaling up, the Cl_int_ value to obtain Cl_int per whole liver (g)/bw(kg)_, the cell concentration in liver, the average liver weight and average body weight parameters were used (Table 1) [29]. The value for hepatic clearance (Cl_H_) was calculated as follows [28]:

$$Cl_{H}=\frac{Cl_{\mathrm{int}liver/bw}*fu*Q_{plasma}}{(Cl_{\mathrm{int}liver/bw}*fu)+Q_{plasma}}$$

where the flow rate of Q_plasma_ = Q_H_* plasma/blood ratio. To calculate Cl_H_, the hepatic flow rate, plasma/blood ratio (Table 1) and fu = 1 values were used [30]. (*Q_H_* is the hepatic blood flow, while *fu* is the unbound fraction of the compound.) The hepatic extraction ratio was defined as *E = Cl_H_/Q_H_*, and the bioavailability (%) was *F = (1 − E)* * 100 [28,31,32,33].

### 2.5. Statistical Analysis

Data of normal distribution and homogeneity of variances were evaluated with one-way ANOVA and Tukey’s post hoc test using the R version 4.0.4 software package (Vienna, Austria, 2021). The results were expressed as means ± SD, and differences between obtained results were determined to be significant if *p* was <0.05 (* *p*  <  0.05, ** *p*  <  0.01, *** *p*  <  0.001). Every measurement was repeated at least in triplicate with 5% interday and intraday precision.

## 3. Results

**Figure 2:**
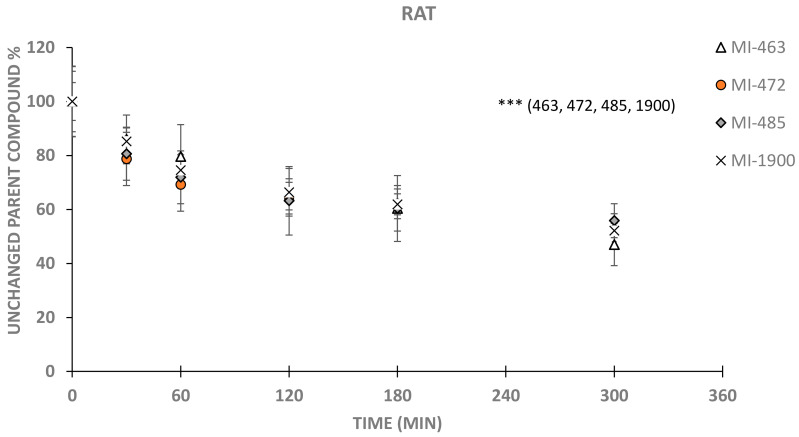
Time course of depletion of inhibitors MI-463, MI-472, MI-485 and MI-1900 in rat primary hepatocytes (5 × 10^5^/mL) using 0, 30, 60, 120, 180 and 300 min sampling times. Relative percentage values of unchanged parent compounds were calculated by dividing the concentrations of the inhibitor measured at a given time point by mean starting MI concentration in the treatment solution (mean ± SD; n = 3–9/group). Asterisks indicate significant differences compared to starting values of the indicated inhibitors in parentheses at 300 min (*** *p* < 0.001).

In rat hepatocytes, all inhibitors were significantly depleted even at 60 min sampling time (p_MI-463, 60 min_ = 0.02755; p_MI-472, 60 min,_ p_MI-485, 60 min_ and p_MI-1900, 60 min_ < 0.001). It was also ascertained that conversions of the inhibitors were elevated to a greater extent independently of the structural differences in the 3-APhA derivatives (*p* < 0.001 in each case) at 120, 180 and 300 min. Comparing the depletion rates of 3-APhA derivatives with various structural moieties showed that they did not differ from each other significantly at 300 min sampling time (*p* values were in the range of 0.3367 and 1); however, they were depleted significantly after 5 h incubation with hepatocytes (*p* < 0.001) (Figure 2). It is also interesting to note that although not much difference in hepatic extraction ratios (E) for MI compounds was predicted, the 0.3 cut-off value between low and intermediate extraction drugs assigned MI-472 and MI-485 as intermediate, while MI-463 and MI-1900 were assigned as low-extraction drugs.

**Figure 3:**
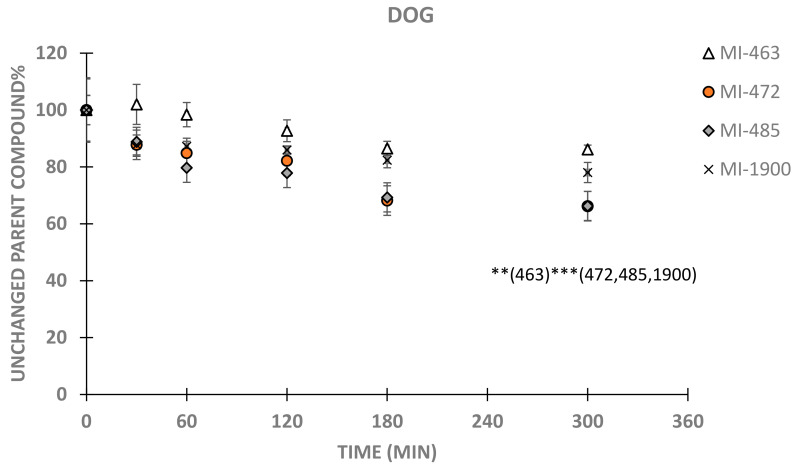
Time course of depletion of inhibitors MI-463, MI-472, MI-485 and MI-1900 in dog primary hepatocytes (5 × 10^5^/mL) using 0, 30, 60, 120, 180 and 300 min sampling times. Relative percentage values of unchanged parent compounds were calculated by dividing the concentrations of the inhibitor measured at given time point by mean starting MI concentration in the treatment solution (mean ± SD; n = 3–9/group). Asterisks indicate significant differences compared to starting values of the indicated inhibitors in parentheses at 300 min (** *p* < 0.01, *** *p* < 0.001).

In dog hepatocytes, MI-472, MI-485 and MI-1900 were significantly depleted even at 30 min and 60 min sampling times (p_MI-472, 30 min_ = 0.0412; p_MI-485, 30 min_ and p_MI-1900, 30 min_ < 0.001; p_MI-472, 60 min_ = 0.0014; both p_MI-485, 60 min_ and p_MI-1900, 60 min_ < 0.001). MI-463 content slowly decreased from 180 min (p_MI-463, 180 min_ = 0.0054 and p_MI-463, 300 min_ = 0.0042), in contrast to accelerated transformation rates of MI-485 and MI-1900, which could suggest that minor structural variations present in the 3-APhA derivatives could cause different depletion rates observed in dog hepatocytes (Figure 3). Due to the slow decomposition of the MI-463, significant differences in depletion rates could be seen at 300 min sampling point between MI-463 and the other inhibitors (MI-472, MI-485 and MI-1900, <0.001 in each case).

**Figure 4:**
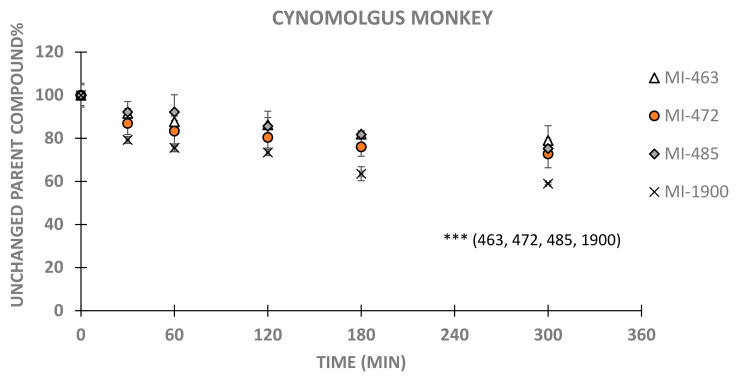
Time course of depletion of inhibitors MI-463, MI-472, MI-485 and MI-1900 in macaque primary hepatocytes (5 × 10^5^/mL) using 0, 30, 60, 120, 180 and 300 min sampling times. Relative percentage values of unchanged parent compounds were calculated by dividing the concentrations of the inhibitor measured at given time point by mean starting MI concentration in the treatment solution (mean ± SD; n = 3–9/group). Asterisks indicate significant differences compared to starting values of the indicated inhibitors in parentheses (*** *p* < 0.001).

Cynomolgus monkey hepatocytes could convert all inhibitors significantly after 120 min treatments (p_MI-463, 120 min_ < 0.001; p_MI-472, 120 min_ = 0.0109; p_MI-485, 120 min_ = 0.0015; p_MI-1900, 120 min_ < 0.001). It was also observed that MI-1900 was depleted at the highest speed, and even from 60 min the decomposition was much higher compared to initial values (Figure 4). No chemical structure-related differences were observed in cynomolgus macaque hepatocytes at 300 min sampling time in the case of MI-463, MI-472, MI-485 and MI-1900 (*p* values ranged between 0.06 and 1).

Interspecies comparisons of depletion rates of 3-APhA derivatives at 300 min proved that MI-463 decomposed differently in rat hepatocytes compared to that in dog and cynomolgus macaque (*p* < 0.001); however, there were no differences between dog and cynomolgus macaque samples (*p* = 0.964). This interspecies variation was also seen in the case of MI-472 (*p* _rat-dog_ = 0.0106; *p* _rat-monkey_ < 0.001; *p* _dog-cynomolgus monkey_ = 0.9713) and MI-1900 (*p* _rat-dog_ < 0.001; *p* _rat-monkey_ = 0.4974; *p* _dog-cynomolgus monkey_ = 0.0128); however, MI-485 did not decompose differently in hepatocytes of the used mammalian species (*p* _rat-dog_ = 0.1341; *p* _dog-monkey_ = 1.000; *p* _rat-monkey_ = 0.2269).

Some interspecies differences in the intrinsic clearance of inhibitors were observed between rat, dog and monkey hepatocytes (Table 2). Rat cells were the most active in biotransformation of all of these compounds (Cl_int_: 31.9–37.8 mL/min/kg), whereas the metabolizing capacity of monkey hepatocytes was the lowest (Cl_int_: 6.6–12.0 mL/min/kg), except for MI-1900, which was eliminated somewhat faster than in dog cells (Cl_int_: 16.1 vs. 10.5 mL/min/kg, respectively). On the basis of hepatic extraction ratios, all four 3-APhA derivatives were designated as low-extraction drugs in monkey cells, while in rat and dog hepatocytes, MI-463 and MI-1900 were low-extraction drugs, and MI-472 and MI-485 were considered to be intermediate-extraction ones. The bioavailability values ranged between 65.7% and 87.9%, predicting low hepatic first-pass effect.

## 4. Discussion

Infections with SARS-CoV-2 caused the COVID-19 pandemic, leading to a significant global health threat. Emerging findings support that entry of SARS-CoV-2 into host cells also highly depends on serine protease activities and that the suppression of TTSP-related cleavage of viral surface glycoprotein hinders fusion of the virus with host cell membranes.

In vitro studies also confirmed that the 3-APhA-derived inhibitors such as MI-432 and MI-1900 could successfully lower viral titers in Calu-3 cells infected with SARS-CoV-2 [27] in a dose-dependent manner, most likely through TMPRSS2-mediated cleavage occurring at the S2′ site. Inhibitor MI-485, with improved selectivity against thrombin and factor Xa, showed inhibition of matriptase-dependent H9N2 influenza virus replication in Madin-Darby canine kidney (MDCK) cells [15].

Four structurally related 3-APhA-derived inhibitors with an N-terminal dimethoxy-substituted biphenyl-3-sulfonyl group were investigated in this work. In our previous studies, we proved that these 3-APhA-derived inhibitors, such as MI-1900 and its structurally related analogues, did not exert any cytotoxic properties, and it was also found that the investigated inhibitors MI-432, MI-463, MI-482 and MI-1900 did not bind to human serum albumin to a great extent and did not influence the activity of cytochrome P 450 isoenzymes (CYP)1A2, 2C9 and 2C19. However, they could modulate human CYP3A4 function and cynomolgus monkey CYP1A2 [34,35].

The present study examined the pharmacokinetic behavior of 3-APhA-derived protease inhibitors MI-463, MI-472, MI-485 and MI-1900 with potential antiviral effects in primary hepatocytes derived from rat, dog and cynomolgus monkey. Based on previous enzyme kinetic measurements, MI-463 and MI-1900 acted as competitive inhibitors of TMPRSS2 and matriptase with very low, nanomolar Ki values, and MI-485 could also suppress matriptase activity with Ki lower than 5 nM. To characterize the pharmaco-toxicological properties of these compounds, cell-based assays were used without excessive sacrifice of experimental animals, in good agreement with animal welfare policies, and at the same time, extrapolatable in vitro PK data were obtained. Primary hepatocytes are widely accepted as a suitable model for studying in vitro pharmacokinetic properties of newly synthesized or repurposed compounds, including CYP inducibility and biotransformation rates in the liver, compared to tumorigenic cellular systems such as human hepatoma cell line HepG2 [36,37,38]. Hence, they could serve as suitable tools for research related to the hepatic metabolism of MI compounds in rat, dog and cynomolgus monkey.

It was ascertained that in rat hepatocytes, the depletion rates of the investigated MI compounds were higher than in dog or monkey cells and were similar to each other independently of their chemical structural differences. Nevertheless, significant interspecies differences in depletion rates of MI compounds were demonstrated. The piperidine-derived MI-463 with C-terminal cyclohexyl urea moiety showed pronounced stability against biotransformation in dog samples. In contrast, elevated depletion rates of this inhibitor were observed in rat and macaque hepatocytes.

The fact that the intrinsic clearance values for these compounds were lower than the rates of hepatic blood flow means that drug metabolizing capacity of the liver rather than the liver blood flow is expected to primarily determine the elimination of MI compounds. The interspecies differences in hepatic extraction properties of MI derivatives anticipate different systemic exposures of rat, dog and monkey at the same dose level. Furthermore, 3-APhA derivatives were proven to be low- or intermediate-extraction drugs with E < 0.34, which means that slight change in drug metabolizing ability is expected to highly influence the hepatic clearance, whereas variations in hepatic blood flow will poorly affect the clearance of these compounds. The bioavailability values were predicted to be higher than 65% for all 3-APhA derivatives, anticipating that hepatic metabolism will poorly decrease the systemic exposure of these drugs.

Several cellular and animal models have been developed for investigation of virus biology under experimental conditions. Due to their low cost, small size and rapid breeding, mouse models (transgenic mouse expressing hACE2, hACE2-transduced mouse, mouse adapted SARS-CoV-2 strain and hACE2 humanized mouse) and hamster models have been used with some limitations, such as lack of severity of the respiratory symptoms. The cynomolgus macaque model was reported to be suitable for studying the pathology and immune responses of the SARS-CoV-2 infection due to the close genetic similarity of cynomolgus macaques to humans; however, their limited availability, the ethical concerns, and the high cost related to the need for complex husbandry restrict their frequent usage in preclinical COVID-19 investigations [39,40,41,42,43]. It was also reported that liver metabolism in cynomolgus monkeys shows strong resemblance to that in humans, including the CYP expressions and drug uptake of hepatocytes, thereby allowing better prediction of human in vivo intrinsic hepatic clearance [44,45,46]. Cynomolgus monkey hepatocytes could metabolize the piperazine-derived MI-1900 rapidly. That is in good agreement with our previous experimental findings pointing out that previously tested TTSP inhibitors could be metabolized significantly even upon 60 min exposure to mammalian hepatic microsomal CYP isoenzymes [34,35].

The One Health movement includes the screening and development of novel drugs for the prevention and treatment of human and animal diseases (www.onehealthinitiative.com). The findings of these in vitro studies can contribute to the practical application of integrated multidisciplinary approaches in both human medicine and veterinary practice. In vitro studies such as our primary hepatocytes-based depletion assays facilitate the selection of suitable drug candidates and thus provide PK support prior to in vivo studies and indirectly contribute to preventing severe adverse effects in laboratory animals and later in clinical trials. In our further experiments, additional PK parameters for these protease inhibitors are planned to be elucidated, including the protein binding properties and potential modulatory effects on isoenzyme activity, in addition to elucidation of their safety profiles, such as the selectivity of these inhibitors against blood clotting factors.

## 5. Conclusions

In conclusion, the current study also confirmed that mammalian cellular studies can be applied to predict important PK parameters of 3-APhA-type antiviral drug candidates prior to preclinical animal studies using depletion rates of the inhibitors without preliminary animal sacrifice. Differences in metabolic rates of the selected inhibitors could mainly be seen in dog and cynomolgus monkey hepatocytes, of which the latter possesses very strong similarity in biotransformation pattern to humans, whereas the rat hepatocyte-mediated depletion was accelerated and almost independent of the chemical structures of MI compounds. Among the investigated inhibitors, MI-1900 with piperazine ring was depleted faster in macaque hepatocytes compared to other derivatives having a piperidine unit, suggesting that different types of heterocyclic rings on the C terminal might influence the decomposition rates. These results may suggest that further modification of these inhibitors on the C-terminal ureido moieties could improve their PK parameters, such as Cl_int_, CL_H_, E_H_ and bioavailability.

## Figures and Tables

**Table 1:** Physiological parameters for calculation of clearance values [30].

Parameter	Rat	Dog	Monkey
Number of hepatocytes in liver (×10^6^ cells/g liver)	117	215	114
Liver weight (g)	10	320	150
Body weight (kg)	0.25	10	5
Liver blood flow (mL/min/kg)	55.2	30.9	43.6
Plasma/blood ratio	0.58	0.57	0.61

**Table 2:** Summary of rat, dog and macaque pharmacokinetic parameters of inhibitors. F means bioavailability. Cl _int_ = intrinsic clearance, Cl_H_ = hepatic clearance values, E_H_ = hepatic extraction ratio.

	Cl_int_ (mL/min/kg)	Predicted Cl CL_H_	Extraction Ratio E_H_	F (%)	Classification
MI-463					
Rat	31.87	15.97	0.289	71.07	Low-extraction drug
Dog	19.487	9.25	0.299	70.06	Low-extraction drug
Macaque	6.58	5.27	0.121	87.91	Low-extraction drug
MI-472					
Rat	35.58	16.85	0.305	69.47	Intermediate-extraction drug
Dog	26.69	10.61	0.343	65.66	Intermediate-extraction drug
Macaque	12.00	8.27	0.190	87.91	Low-extraction drug
MI-485					
Rat	37.78	17.33	0.314	68.60	Intermediate-extraction drug
Dog	22.75	9.93	0.321	67.87	Intermediate-extraction drug
Macaque	11.40	7.98	0.183	81.69	Low-extraction drug
MI-1900					
Rat	33.76	16.43	0.298	70.23	Low-extraction drug
Dog	10.46	6.56	0.212	78.76	Low-extraction drug
Macaque	16.10	10.03	0.230	76.99	Low-extraction drug

## Data Availability

All raw data supporting the results of the present study can be obtained from the corresponding author upon reasonable request.
